# Relationship of fluconazole prophylaxis with fungal microbiology in hospitalized intra-abdominal surgery patients: a descriptive cohort study

**DOI:** 10.1186/s13054-014-0590-1

**Published:** 2014-10-29

**Authors:** Marya Zilberberg, Hsing-Ting Yu, Paresh Chaudhari, Matthew F Emons, Nikhil Khandelwal, Andrew F Shorr

**Affiliations:** EvidMed Research Group, LLC, PO Box 303, Goshen, MA 01032 USA; School of Public Health and Health Sciences, University of Massachusetts, Amherst, MA USA; Cerner Research, 600 Corporate Pointe, Suite 320, Culver City, CA 90230 USA; Astellas Scientific and Medical Affairs, Inc., 1 Astellas Way, Northbrook, IL 60062 USA; Division of Pulmonary and Critical Care Medicine, Washington Hospital Center, 110 Irving Street, N.W. 2A-64B, Washington, DC USA

## Abstract

**Introduction:**

Historically, *Candida albicans* has represented the most common cause of candidemia. However, the proportion of bloodstream infections due to non-albicans *Candida* species has increased. Because of the risk for candidemia in intra-abdominal surgical patients, some experts advocate the use of fluconazole prophylaxis. The impact of this practice on the distribution of *Candida* species isolated in breakthrough fungal infections in this population is unknown. We examined the association of fluconazole prophylaxis with the distribution of *Candida* species in intra-abdominal surgery patients.

**Methods:**

We retrospectively identified cases with a positive blood culture (BCx) for *Candida* among hospitalized adult intra-abdominal surgery patients between July 2005 and October 2012. Distribution of *Candida* species isolated represented our primary endpoint. Qualifying surgical cases were determined based on a review of discharge International Classification of Diseases, Ninth Revision, Clinical Modification (ICD-9-CM) codes. Patients receiving low-dose fluconazole prior to the positive BCx with a known indication for prophylaxis including neutropenia, ICU exposure or history of organ transplantation were classified as prophylaxis. Appropriateness of fungal treatment was determined by the timing and selection of antifungal agent based on fungal isolate.

**Results:**

Among 10,839 intra-abdominal surgery patients, 227 had candidemia. The most common *Candida* species isolated was *C. albicans* (*n* = 90, 39.6%) followed by *C. glabrata* (*n* = 81, 35.7%) and *C. parapsilosis* (*n* = 38, 16.7%). Non-albicans *Candida* accounted for 57.7% of isolates among the 194 non-prophylaxis patients and 75.8% among the 33 prophylaxis patients (*P* = 0.001). *C. glabrata,* the most common non-*C. albicans* species, was more prevalent than *C. albican*s in persons given prophylaxis, but not in those without prophylaxis. A total of 63% of those with candidemia were treated inappropriately based on the timing and selection of antifungal administration.

**Conclusions:**

Selection pressure from fluconazole prophylaxis in at-risk surgical patients may be associated with a drift toward fluconazole-resistant species in subsequent candidemia. Tools are needed to guide appropriate treatment through the prompt recognition and characterization of candidemia.

**Electronic supplementary material:**

The online version of this article (doi:10.1186/s13054-014-0590-1) contains supplementary material, which is available to authorized users.

## Introduction

With crude mortality approaching 40%, candidemia continues to take a high toll among certain groups of hospitalized patients [1-5]. In the past, the species most frequently responsible for candidemia and invasive *Candida* infections was *Candida albicans* [1]. Over time, however, there has been a shift in the epidemiology of invasive *Candida* infection, with an increasing proportion being due to species other than *C. albicans* [2,3].

Invasive *Candida* is of particular concern in specific populations. Intra-abdominal surgery is a well-recognized risk factor for invasive fungal infection [6,7]. The initial choice of an antifungal is critical in the setting of suspected candidemia, as it is strongly associated with the outcomes. Although well-established evidence-based clinical guidelines help in making correct choices, efforts to ensure appropriate and timely initial antifungal therapy have been complicated by the shifting microbiology and antifungal susceptibility patterns. To address this uncertainty, the guidelines recommend empiric therapy with an echinocandin to treat suspected candidemia in the setting of critical illness, as well as in neutropenia with fluconazole prophylaxis, where non-*C. albicans* isolates are more likely [8]. Unfortunately, candidal microbiology and thus initial coverage decisions are less clear in the setting of intra-abdominal surgery. One factor that may be driving shifts in candidal populations and antifungal susceptibility among these patients is fluconazole prophylaxis, which although controversial is used nevertheless. The concern stems from several studies in nonsurgical cohorts that have detected an association between fluconazole prophylaxis and increased infection with *Candida glabrata* and *Candida krusei*, reflecting the impact of selection pressure with fluconazole prophylaxis [3,9-12]. However, it is unclear whether such selection pressure with a drift toward azole-resistant species is a factor among patients undergoing intra-abdominal surgery who develop candidemia.

The primary aim of our study was to examine whether there is an association between fluconazole prophylaxis and the distribution of *Candida* species found in subsequent breakthrough candidemia among intra-abdominal surgery patients. We also assessed the appropriateness of empiric antifungal therapy among this group of patients with candidemia based on timing, dose, and susceptibility.

## Methods

### Study design and data source

We conducted a retrospective multicenter cohort study using the Cerner *Health Facts*® database. *Health Facts* is a de-identified database built from participating hospitals’ comprehensive medical records, including time-stamped medication orders and laboratory/microbiology data, admission, and billing information from affiliated patient care locations. Data were included from 97 US hospitals with diversity of geographic location, bed size, and teaching status. Cerner Corporation (Kansas City, MO, USA) has established Health Insurance Portability and Accountability Act-compliant operating policies and procedures using statistical methods for de-identification. With the use of an existing de-identified database, institutional review board oversight was deemed inapplicable under Health and Human Services 45 CFR 46.101 (a) (4).

### Cohort selection and study definitions

We included all adult (age ≥18 years old) hospitalized patients discharged between July 2005 and October 2012 with International Classification of Disease, Ninth Revision, Clinical Modification codes for primary or secondary diagnosis of intra-abdominal infection and an invasive abdominal surgery (see Additional file 1) [13]. Patients were included if they had candidemia defined by at least one positive blood culture (BCx) for *Candida* and no BCx with other fungal genera. If patients had more than one candidemia episode during the study window, only the first episode was evaluated for inclusion in the cohort. For purposes of classification and time reference, the draw time of the first positive BCx for *Candida* was used.

Fluconazole prophylaxis was defined as having: a low dose of fluconazole (200 to 400 mg) prior to positive BCx for *Candida* with no prior order for a loading dose of fluconazole or other therapeutic antifungal agent; and a known indication for prophylaxis including neutropenia, ICU exposure, or history of organ transplantation. A small portion (approximately 8% of the cohort) required manual assignment by a clinical pharmacist, who also took other available data elements into consideration (dosage, frequency, and timing with respect to other antifungals ordered).

Appropriate treatment was defined as an order for a loading or therapeutic dose of a qualifying antifungal agent from 96 hours before through 24 hours after positive BCx for *Candida*. The antifungal agents evaluated included azoles, echinocandins, and amphotericin B compounds. Inappropriate treatment was defined as: no antifungal treatment; no order of a qualifying antifungal agent for a therapeutic dose from 96 hours before through 24 hours after positive BCx for *Candida*; or cultures grew *C. glabrata* or *C. krusei* and the patient was treated only with fluconazole. Treatment appropriateness was classified as indeterminate for other scenarios (for example, patients with orders for a therapeutic antifungal dose more than 96 hours prior to positive BCx for *Candida*).

Time to surgery was defined by calendar days from the admission date based on the first surgical procedure of interest. Encounters were classified as urgent emergent if the admission type was coded urgent/emergent or the source was the emergency room or trauma center. Bacteremia required at least one positive BCx for bacteria (excluding common skin contaminants) at any time during the hospital encounter. Baseline laboratory values were selected as the first value after admission. Respiratory failure prior to index culture was defined based on mechanical ventilation International Classification of Disease, Ninth Revision, Clinical Modification procedure codes and arterial blood gas values; and cardiac dysfunction was indicated by orders for intravenous pressors. ICU exposure was defined based on pharmacy, microbiology, and laboratory care settings. Comorbid conditions were defined by primary or secondary International Classification of Disease, Ninth Revision, Clinical Modification codes during or within 12 months prior to the hospital encounter.

### Statistical analyses

Mean and standard deviation were reported for continuous variables, and frequency and percentage were reported for categorical variables. Differences between categorical variables were assessed via chi-squared or Fisher’s exact tests, while those in the continuous variables were examined using the Wilcoxon rank-sum test. The level of statistical significance was set at a two-sided alpha value of 5%. Analyses were performed in SAS version 9.3 (SAS Institute, Cary, NC, USA).

## Results

### Study population characteristics

**Figure 1:**
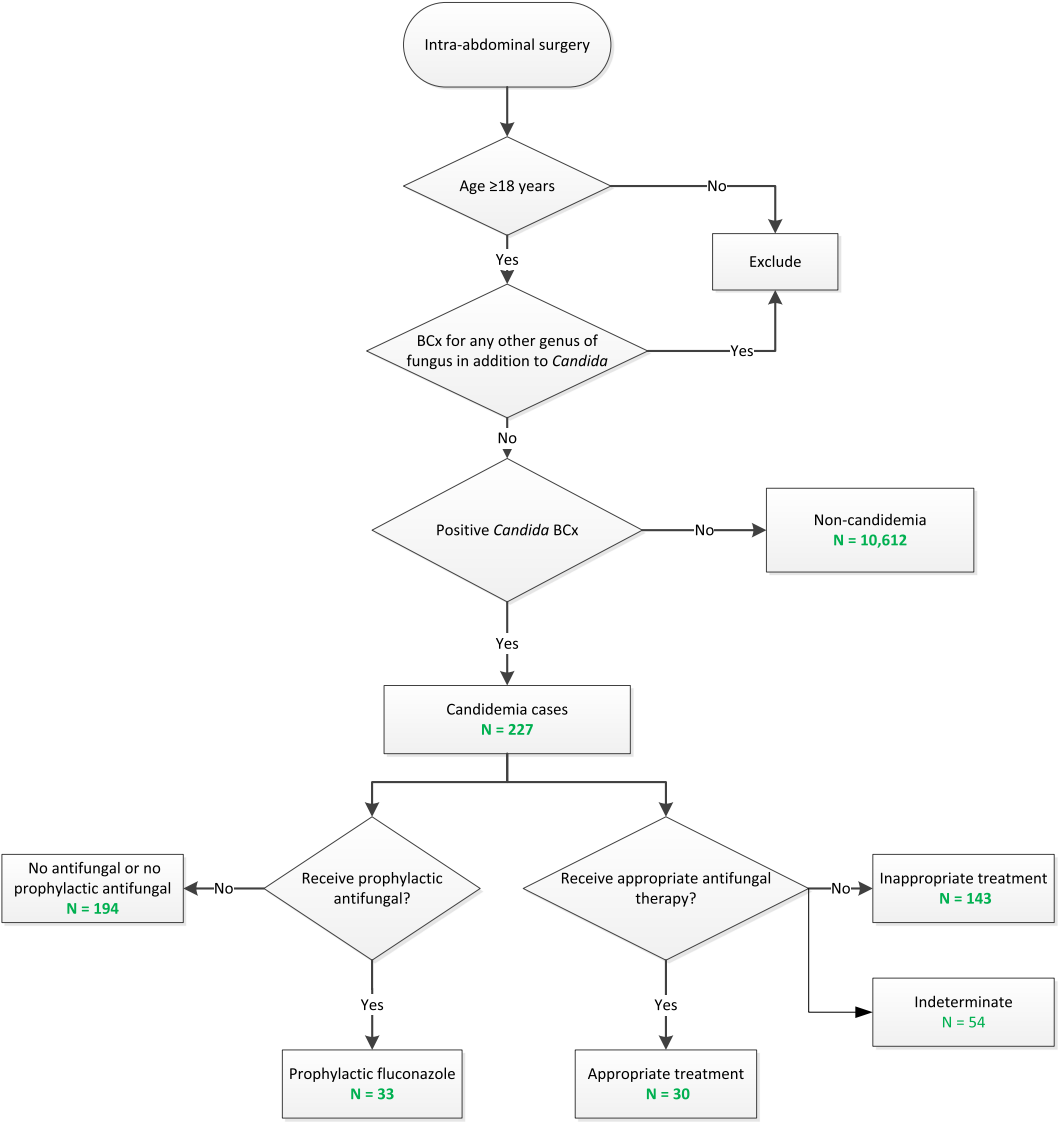
**Study population and groups.** BCx, blood culture.

A total of 10,839 patients who underwent intra-abdominal surgery were identified, and 227 patients (2.1%) had candidemia (Figure 1), of whom 33 (14.5%) had undergone fluconazole prophylaxis (Table 1). Among the 227 candidemia patients the mean age was 62.4 years, approximately one-half were male, and 74% were Caucasian. Less than 2% of the study cohort had evidence of antifungal therapy within 30 days prior to admission. The majority of candidemia patients had their positive BCx for *Candida* >96 hours after admission. Approximately one-half of the study population had the first surgical procedure of interest within 2 days of admission, but nearly one-half had the first surgery 3 days or more after admission. Illness severity in the entire cohort was high, with 54% of the cohort being treated with vasopressors and 68% having respiratory failure prior to positive BCx for *Candida*. Sixty percent of patients were treated in an ICU and 40% of the cohort had bacteremia at some point during the hospital stay.

**Table 1:** **Patient demographic and encounter characteristics**

**Variable**	**All candidemia**	**Fluconazole prophylaxis**	**No prophylaxis**	***P*** **value** ^**a**^
	**(** ***n*** **= 227)**	**(** ***n*** **= 33)**	**(** ***n*** **= 194)**	
Age (years)	62.4 (15.0)	61.7 (13.7)	62.5 (15.2)	0.6188
Female gender	118 (52.0%)	20 (60.6%)	98 (50.5%)	0.2835
Race				
Caucasian	167 (73.6%)	24 (72.7%)	143 (73.7%)	0.4575
African American	45 (19.8%)	9 (27.3%)	36 (18.6%)	
Asian	2 (0.9%)	0 (0.0%)	2 (1.0%)	
Other known	9 (4.0%)	0 (0.0%)	9 (4.6%)	
Unknown	4 (1.8%)	0 (0.0%)	4 (2.1%)	
Admission source				
Hospital/other care facility	35 (15.4%)	5 (15.2%)	30 (15.5%)	0.1363
SNF/NH	3 (1.3%)	0 (0.0%)	3 (1.5%)	
Emergency room	102 (44.9%)	21 (63.6%)	81 (41.8%)	
Other	78 (34.4%)	7 (21.2%)	71 (36.6%)	
Unknown	9 (4.0%)	0 (0.0%)	9 (4.6%)	
Urgent/emergent admission	142 (62.6%)	28 (84.8%)	114 (58.8%)	
Charlson Comorbidity Index score	2.1 (2.4)	2.4 (2.9)	2.0 (2.3)	0.9624
Comorbid conditions				
Diabetes	56 (24.7%)	10 (30.3%)	46 (23.7%)	0.4168
Hypertension	83 (36.6%)	10 (30.3%)	73 (37.6%)	0.4192
Coronary artery disease	29 (12.8%)	7 (21.2%)	22 (11.3%)	0.1163
Heart failure	32 (14.1%)	6 (18.2)	26 (13.4%)	0.4657
Prior stroke/TIA	12 (5.3%)	5 (15.2%)	7 (3.6%)	0.0062
COPD/bronchiectasis	36 (15.9%)	6 (18.2%)	30 (15.5%)	0.6927
Encounter events				
Bacteremia during index encounter	94 (41.4%)	18 (54.5%)	76 (39.2%)	0.0975
ICU exposure during index encounter	135 (59.5%)	30 (90.9%)	105 (54.1%)	<0.0001
Respiratory failure prior to positive BCx for *Candida*	154 (67.8)	25 (75.8)	129 (66.5)	0.2923
IV vasopressor prior to positive BCx for *Candida*	123 (54.2)	24 (72.7)	99 (51.0)	0.0207
Baseline laboratory parameters				
WBC (k/mm^3^)	12.8 (8.6)	13.2 (11.2)	12.7 (8.1)	0.597
Neutropenia (ANC <500 cells/mm^3^) any time	27 (11.9%)	9 (27.3%)	18 (9.3%)	0.0181
Blood glucose (mg/dl)	146.5 (72.2)	145.4 (66.4)	146.7 (73.3)	0.8315
eGFR (ml/minute/1.73 m^2^)	65.4 (45.8)	69.8 (69.0)	64.6 (40.7)	0.8255
Time from presentation to positive BCx for *Candida* draw				
< 48 hours	16 (7.0%)	1 (3.0%)	15 (7.7%)	0.1914
48 to 96 hours	12 (5.3%)	0 (0%)	12 (6.2%)	
> 96 hours	199 (87.8%)	32 (97.0%)	167 (86.1%)	
Time from presentation to initial procedure of interest				
On the day of admission	70 (30.8%)	10 (30.3%)	60 (30.9%)	0.5945
1 to 2 days	44 (19.4%)	7 (21.2%)	37 (19.1%)	
≥ 3 days	113 (49.8%)	16 (48.5%)	97 (50.0%)	
Antifungal therapy within 30 days prior to admission	3 (1.3%)	1 (3.0%)	2 (1.0%)	0.3525

Data presented as mean (standard deviation) or number (percentage). ANC, absolute neutrophil count; BCx, blood culture; COPD, chronic obstructive pulmonary disease; eGFR, estimated glomerular filtration rate; IV, intravenous; SNF/NH, skilled nursing facility/nursing home; TIA, transischemic attack; WBC, white blood cell count. ^a^ *P* value for the comparison between fluconazole prophylaxis and nonprophylaxis.

When comparing 33 prophylaxis patients (distributed across 18 institutions) with 194 patients who had not undergone fluconazole prophylaxis (distributed across 47 institutions), illness severity was generally higher in the prophylaxis group as evidenced by the need for vasopressors (73% vs. 51%, *P* = 0.02) and by having respiratory failure (76% vs. 67%, *P* >0.05) or hematologic dysfunction (58% vs. 33%, *P* = 0.007) prior to positive BCx for *Candida*. Occurrence of bacteremia was also more frequent among patients who received fluconazole prophylaxis compared with those who did not, but this difference did not reach statistical significance (55% vs. 39%, respectively, *P* = 0.098).

The distribution of *Candida* species found in positive BCx for *Candida* is presented in Table 2. Overall, the most common *Candida* species was *C. albicans*, followed by *C. glabrata* and *C. parapsilosis.* However, within the prophylaxis group, *C. glabrata* was the most common isolate, followed by *C. albicans* and *C. parapsilosis*. The proportion of non-*C. albicans* species was 76% in the fluconazole prophylaxis group compared with 58% in the nonprophylaxis group (*P* = 0.001).

**Table 2:** **Distribution of** ***Candida*** **species based on positive blood culture for total candidemia, prophylaxis and no prophylaxis subgroups**

**Candida species**	**Candidemia**		**Fluconazole prophylaxis**		**No prophylaxis**	
	**(** ***n*** **= 227)**		**(** ***n*** **= 33)**		**(** ***n*** **= 194)**	
	***n***	**%**	***n***	**%**	***n***	**%**
Albicans	90	39.65	8	24.24	82	42.27
Glabrata	81	35.68	16	48.48	65	33.51
Parapsilosis	38	16.74	6	18.18	32	16.49
Tropicalis	14	6.17	2	6.06	12	6.19
Lusitaniae	3	1.32	1	3.03	2	1.03
Dubliniensis	2	0.88	0	0.00	2	1.03
Krusei	2	0.88	1	3.03	1	0.52
Guilliermondii	1	0.44	0	0.00	1	0.52
Other	1	0.44	0	0.00	1	0.52

### Appropriateness of therapy

One hundred and forty-three patients (63%) met the criteria for inappropriate therapy based on the timing, dose, and selection of antifungal therapy (Figure 1). Only 30 patients (13%) could be classified as having received appropriate treatment, with antifungal therapy for the remaining patients categorized as indeterminate.

## Discussion

Our study has confirmed that *C. albicans* was the most common species isolate among patients with intra-abdominal surgery and candidemia (39.6%), followed closely by *C. glabrata* (35.7%). Overall, non-*C. albicans* species accounted for more than one-half of all isolates and three-quarters of isolates among those who had fluconazole prophylaxis. Most alarmingly, based on timing and selection of empiric coverage, 63% of candidemia patients received treatment categorized as inappropriate based on conservative criteria.

There are several implications to the distribution of non-*C. albicans* species found among patients with prophylaxis. Admittedly, patients with prophylaxis who developed non-*C. albicans* candidemia were more severely ill than those without prophylaxis, as evidenced by their need for vasopressors and mechanical ventilation, as well as by their higher likelihood of having bacteremia. One could hypothesize that such patients are more likely to have had prior treatment, and not just prophylaxis, with fluconazole, which may have served as the stimulus for development of resistant species. Our data did not allow us to explore their prior exposure to fluconazole as treatment, an important question to address in future studies. However, the increase in resistant species among patients with prophylaxis also probably points to the selection pressures inherent in antimicrobial prophylaxis in this surgical cohort of patients with intra-abdominal infections, which is not well documented in prior studies. The risks and benefits of fluconazole prophylaxis in this group, although controversial, thus need to continue to be evaluated carefully, given its propensity to drive a switch to resistant species in this deadly disease. Importantly, when faced with a patient who has received prophylaxis, a clinician must consider that such a patient’s risk for a resistant candidemia may be considerably elevated and factor this information into his/her empiric treatment decisions.

Controversy remains regarding antifungal prophylaxis among intra-abdominal surgery patients. Research efforts to resolve these questions are impeded by the need for large sample sizes to demonstrate adjusted differences in clinical outcomes. Changing *Candida* epidemiology further complicates research and renders some prior studies obsolete. While shown effectively to decrease the incidence of fungal infection in high-risk, critically ill surgical patients [9,14-16], fluconazole prophylaxis does not appear to improve their survival [9,16]. These studies indicate that the benefit to the patient of the decision to use prophylaxis may not exceed the risk of driving the escalation of resistance to existing antifungal treatments. Our data indicate that although only a small proportion of this surgical population received prophylaxis, their risk of a resistant candidal species was high.

While earlier studies reported that non-*C. albicans* species accounted for one-half or fewer of isolated organisms [17,18], later research uncovered an increase in cases of candidemia involving non-*C. albicans* [2,3]. We observed a similarly increased proportion of non-*C. albicans* isolates, most pronounced among patients receiving prophylaxis with fluconazole. This switch in species is concerning, since non-*C. albicans* species, and in particular *C. glabrata*, exhibit greater resistance to fluconazole and are associated with worse outcomes when compared with *C. albicans* [19].

The association of increasingly resistant candidal species causing candidemia and their associated worsened outcomes may be explained by the difficulty of targeting initial empiric therapy amidst the shifting landscape of antifungal susceptibility. Many studies in both candidemia and other serious infections have noted a significant and clinically important rise in the risk of death when the patient does not receive prompt empiric treatment with an agent that covers the culprit pathogen [20-22]. Guidelines for patients with complicated intra-abdominal infections [23] recommend antifungal prophylaxis for healthcare-associated infection and those with severe community-acquired infection, leading with fluconazole. An echinocandin is recommended as initial therapy for critically ill patients, although no guidance is provided with respect to recent fluconazole prophylaxis. The Infectious Disease Society of America Expert Panel favors echinocandins for treating suspected invasive candidiasis in non-neutropenic patients who are moderately or severely ill or had recent azole exposure [8]. Guidelines recommend antifungal agents based on the *Candida* isolate or suspected isolate, but delaying appropriate selection until culture results are available or until the patient is critically ill may be too late [8,23].

More than 60% of our study population failed to receive appropriate empiric therapy based on timing and coverage (the latter in those whose BCx grew non-*C. albicans* species). We chose not to evaluate adjusted mortality based on the appropriateness of treatment due to sample size considerations in the context of the low percentage of patients with evidence of appropriate treatment. The challenges that are leading to inappropriate treatment suggest the need for additional improvements to rapidly recognize and characterize candidemia. Risk stratification algorithms and emerging diagnostics such as β-D-glucan may play a role in the future [24-26].

Our study is subject to a number of limitations. As a retrospective cohort, the study is prone to a number of biases, most prominently selection bias. To mitigate this bias, we included all consecutive patients who met our *a priori* inclusion criteria. Despite the fact that we started out with more than 10,000 surgical patients at risk for candidemia, the final cohort with the infection was relatively small. While it would have been desirable to perform several stratified or adjusted analyses, including those based on dose of fluconazole prophylaxis and year of data with regard to resistance emergence, this limitation precluded such computations. Because physiologic parameters needed to derive such severity of illness scores as the Acute Physiology and Chronic Health Evaluation II score are not available in the study database, we relied on such markers as the need for the ICU, vasopressors, and mechanical ventilation as surrogates. As such, our findings require further exploration in the context of a validated severity of illness system.

Our data may be particularly prone to misclassification of several factors. For example, we used a relatively stringent definition for fluconazole prophylaxis. This was done in order to increase the specificity of the definition. As a consequence, we probably misclassified some patients who did not fit this definition and yet received prophylaxis as not receiving prophylaxis. We chose specificity over sensitivity in order to avoid inflating the magnitude of the differences in the candidal species between the two groups. As a result, the actual differences in the prevalence of potentially azole-resistant species between the groups on and off prophylaxis are probably even greater than what we have observed. On the other hand, it remains possible that at least some of the cases identified as prophylaxis in reality received treatment. However, the stringent nature of our definition of prophylaxis should have minimized such misclassification. The appropriateness of empiric antifungal therapy could not be determined reliably in nearly 25% of the study population based on data elements that were available. However, even in the unlikely event that all of these patients could have been ultimately assigned to the appropriate group, this would not detract from the finding that 63% did not receive appropriate therapy.

A distinct strength of our study, as compared with prior single-center reports, is its multicenter nature, which lends generalizability to our findings.

## Conclusions

In summary, our study provides further evidence of the microbiologic shifts in candidemia among a population of patients who are at particular risk for this infection. To the best of our knowledge, this is the first study to examine the association of fluconazole prophylaxis with the candidal species distribution among intra-abdominal surgery patients. The result of this examination points to a likely role for selection pressures in such prophylaxis patients. In turn, the high prevalence of inappropriate treatment detected in our study is an important reminder for clinicians to consider candidemia in a fitting clinical setting, and to be aware of the factors that drive antifungal resistance. Most importantly, in the era of a widening gap between evolving microbial defenses and our abilities to address them, antifungal prophylaxis practices among intra-abdominal surgical patients require a measured re-evaluation.

## Key messages

- *C. albicans* was the most common fungal isolate among intra-abdominal surgery patients with candidemia, followed closely by *C. glabrata.*
- The percentage of nonalbicans Candida species was disproportionately high among intra-abdominal surgery patients treated with fluconazole prophylaxis.
- The shifting epidemiology of fungal species in intra-abdominal infections and the potential for selection pressure has significant implications to prophylaxis therapy and the empiric treatment of those with suspected infection.
- More than 60% of patients did not receive appropriate antifungal therapy based on timing and selection of antifungal agent.

## Additional file

Additional file 1:
**Is a table presenting the diagnosis of intra-abdominal infection and invasive abdominal surgery by the International Classification of Disease, Ninth Revision, Clinical Modification (ICD-9-CM) code.**

## Abbreviation

BCx: blood culture
