# Supplementary material for: Relationship of fluconazole prophylaxis with fungal microbiology in hospitalized intra-abdominal surgery patients: a descriptive cohort study
Source: Crit Care. 2014 Oct 29;18(5):590. doi: 10.1186/s13054-014-0590-1 (PMC4234884; doi:10.1186/s13054-014-0590-1)
Supplement: Additional file 1: — Is a table presenting the diagnosis of intra-abdominal infection and invasive abdominal surgery by the International Classification of Disease, Ninth Revision, Clinical Modification (ICD-9-CM) code. [file 13054_2014_590_MOESM1_ESM.doc]

Additional file 1: Diagnosis of intra-abdominal infection and invasive abdominal surgery by ICD-9-CM code

| **ICD-9-CM Diagnosis*** | **Description** |
| --- | --- |
| 531.1×, 531.5× | Gastric ulcer with perforation |
| 531.2×, 531.6× | Gastric ulcer with hemorrhage and perforation |
| 532.1×, 532.5× | Duodenal ulcer with perforation |
| 532.2×, 532.6× | Duodenal ulcer with hemorrhage and perforation |
| 533.1×, 533.5× | Peptic ulcer with perforation |
| 533.2×, 533.6× | Peptic ulcer with hemorrhage and perforation |
| 534.1×, 534.5× | Gastrojejunal ulcer with perforation |
| 534.2×, 534.6× | Gastrojejunal ulcer with hemorrhage and perforation |
| 540.0 | Acute appendicitis with generalized peritonitis |
| 540.1 | Acute appendicitis with peritoneal abscess |
| 567.X | Peritonitis |
| 569.5 | Abscess of intestine |
| 569.81 | Fistula of intestine, excl. rectum and anus |
| 569.82 | Ulceration of intestine |
| 569.83 | Perforation of intestine |
| 572.0 | Abscess of liver |
| 575.4 plus (574.0, 574.3, 574.6, 574.8, or 575.0) | Acute cholecystitis |

* In ICD-9-CM, “X” denotes a wildcard that includes a missing digit as well as the range of digits that span from 0 to 9

| **ICD-9-CM Procedure*** | **Description** |
| --- | --- |
| 43.5 | Partial gastrectomy with anastomosis to esophagus |
| 43.6 | Partial gastrectomy with anastomosis to duodenum |
| 43.7 | Partial gastrectomy with anastomosis to jejunum |
| 43.8× | Other partial gastrectomy |
| 43.9× | Total gastrectomy |
| 44.40 | Suture of peptic ulcer, not otherwise specified |
| 44.41 | Endoscopic excision or destruction of lesion or tissue of stomach |
| 44.42 | Local excision of other lesion or tissue of stomach |
| 44.61 | Suture of laceration of stomach |
| 45.6× | Other excision of small intestine |
| 45.7× | Open and other partial excision of large intestine |
| 45.8× | Total intra-abdominal colectomy |
| 45.9× | Intestinal anastomosis |
| 46.0× | Exteriorization of intestine |
| 46.10 | Colostomy, not otherwise specified |
| 46.11 | Temporary colostomy |
| 46.13 | Permanent colostomy |
| 46.20 | Ileostomy, not otherwise specified |
| 46.21 | Temporary ileostomy |
| 46.22 | Continent ileostomy |
| 46.23 | Other permanent ileostomy |
| 46.7× | Other repair of intestine |
| 46.80 | Intra-abdominal manipulation of intestine, not otherwise specified |
| 46.81 | Intra-abdominal manipulation of small intestine |
| 46.82 | Intra-abdominal manipulation of large intestine |
| 46.91 | Myotomy of sigmoid colon |
| 46.92 | Myotomy of other parts of colon |
| 46.93 | Revision of anastomosis of small intestine |
| 46.94 | Revision of anastomosis of large intestine |
| 46.99 | Other (Ileoentectropy) |
| 47.XX | Operations on appendix |
| 50.0 | Hepatotomy |
| 50.12 | Open biopsy of liver |
| 50.13 | Transjugular liver biopsy |
| 50.14 | Laparoscopic liver biopsy |
| 50.19 | Other diagnostic procedures on liver |
| 50.2× | Local excision or destruction of liver tissue or lesion |
| 50.3 | Lobectomy of liver |
| 50.4 | Total hepatectomy |
| 50.5× | Liver transplant |
| 50.6× | Repair of liver |
| 51.02 | Trocar cholecystostomy |
| 51.03 | Other cholecystostomy |
| 51.04 | Other cholecystotomy |
| 51.13 | Open biopsy of gallbladder or bile ducts |
| 51.2× | Cholecystectomy |
| 51.3X | Anastomosis of gallbladder or bile duct |
| 51.4× | Incision of bile duct for relief of obstruction |
| 51.5× | Other incision of bile duct |
| 51.61 | Excision of cystic duct remnant |
| 51.62 | Excision of ampulla of Vater (with reimplantation of common duct) |
| 51.63 | Other excision of common duct |
| 51.69 | Excision of other bile duct |
| 51.7× | Repair of bile ducts |
| 51.81 | Dilation of sphincter of Oddi |
| 51.82 | Pancreatic sphincterotomy |
| 51.83 | Pancreatic sphincteroplasty |
| 51.89 | Other operations on sphincter of Oddi |
| 51.91 | Repair of laceration of gallbladder |
| 51.92 | Closure of cholecystostomy |
| 51.93 | Closure of other biliary fistula |
| 51.94 | Revision of anastomosis of biliary tract |
| 51.95 | Removal of prosthetic device from bile duct |
| 51.99 | Other (Insertion or replacement of biliary tract prosthesis) |
| 52.0× | Pancreatotomy |
| 52.12 | Open biopsy of pancreas |
| 52.22 | Other excision or destruction of lesion or tissue of pancreas or pancreatic duct |
| 52.3 | Marsupialization of pancreatic cyst |
| 52.4 | Internal drainage of pancreatic cyst |
| 52.5× | Partial pancreatectomy |
| 52.6 | Total pancreatectomy |
| 52.7 | Radical pancreaticoduodenectomy |
| 52.80 | Pancreatic transplant, not otherwise specified |
| 52.81 | Reimplantation of pancreatic tissue |
| 52.82 | Homotransplant of pancreas |
| 52.83 | Heterotransplant of pancreas |
| 52.92 | Cannulation of pancreatic duct |
| 52.95 | Other repair of pancreas |
| 52.96 | Anastomosis of pancreas |
| 52.99 | Other (Dilation/repair of pancreatic [Wirsung's] duct by open approach) |
| 54.1× | Laparotomy |
| 54.21 | Laparoscopy |
| 54.4 | Excision or destruction of peritoneal tissue |
| 54.5× | Lysis of peritoneal adhesions |
| 54.92 | Removal of foreign body from peritoneal cavity |
| 54.93 | Creation of cutaneoperitoneal fistula |
| 54.94 | Creation of peritoneovascular shunt |
| 54.95 | Incision of peritoneum |
